# Editorial: Targeted Therapies for Glioblastoma: A Critical Appraisal

**DOI:** 10.3389/fonc.2019.01216

**Published:** 2019-11-12

**Authors:** Shiv K. Gupta, Sani H. Kizilbash, David J. Daniels, Jann N. Sarkaria

**Affiliations:** ^1^Departments of Radiation Oncology, Mayo Clinic, Rochester, MN, United States; ^2^Departments of Oncology, Mayo Clinic, Rochester, MN, United States; ^3^Department of Neurosurgery, Mayo Clinic, Rochester, MN, United States

**Keywords:** GBM, DIPG, diffuse intrinsic pontine glioma, novel therapy, blood-brain barrier, drug delivery, targeting

High grade gliomas including glioblastoma (GBM) in adults and diffuse intrinsic pontine glioma (DIPG) in children are fatal brain tumors with <5% of patients surviving 5 years after initial diagnosis and treatment. Targeted agents hold promise as monotherapy or as sensitizing strategies to improve response to traditional chemo-radiation. However, despite intense research endeavors and numerous clinical trials, no targeted agents have been FDA approved in the past decade. Clonal heterogeneity, acquired or inherent resistance to available therapies, restricted drug delivery, resistant stem-like cells, and immune-evasive properties in these tumors have been subject to intense study. Advances in technology including next-generation sequencing- has allowed comprehensive mapping of genetic alterations such as single nucleotide polymorphism, fusions, and copy number variations, and alterations to the epigenetic landscape including DNA methylation and histone post-translational modifications (PTMs). This approach has led to identification of several therapeutic targets and potential biomarkers, resulting in a number of new investigational treatment modalities. These include inhibitors of signaling, cell cycle and DNA damage repair pathways, and angiogenesis, in addition to ongoing evaluations of novel gene-, viro-, and immuno-therapies. However, as highlighted in the series of articles (referenced) and compiled in this eBook, despite significant progress in understanding pathology and molecular underpinnings, clinical development of novel therapeutics has faced challenges that hinder overall progress.

## Inhibitors of Signaling and Angiogenesis

A number of therapeutic agents targeting growth factor receptors and downstream pathways, cell cycle, epigenetic modulators, angiogenesis, and antitumor immune responses have been tested. Targeting of receptor tyrosine kinases (EGFR, FGFR, PDGFR, and cMET) or their downstream signaling pathways (PI3K/AKT/mTOR and MAPK) using small molecule kinase inhibitors, antibodies, or antibody drug conjugates (ADCs) have been extensively studied (Jain). However, except a few documented cases of response, both kinase inhibitors and monoclonal antibodies or antibody-drug conjugates (ADCs) targeting receptor tyrosine kinases have failed to prolong overall or progression-free survival (PFS) of patients with GBM or DIPG. Adding to the pipeline of signaling inhibitors, Sheng et al., show that the drug importazole, by disruption of interaction between RAN-GTPase and KPNB1, inhibits growth of GBM cells. Therefore, targeting of RAN-GTPase with importazole appears to be a promising strategy for GBM (Sheng et al.). However, analysis of brain pharmacokinetics and *in vivo* efficacy of importazole should be carefully determined to allow further development of importazole as targeted therapy for GBM. Similarly, angiogenesis inhibitors evaluated as monotherapy or in combination therapies in clinical trials of GBM also had no significant benefit in overall survival, although combining bevacizumab with the standard of care led to an increased PFS in subset of patients enrolled in recent clinical trials involving patients with newly diagnosed GBM. Determining predictive biomarkers may help fully harness the benefits of anti-angiogenic agents. Malo et al. report that the antiangiogenic agents potentiate immune responses, which probably leads to the improved PFS in subset of patients on clinical trials of antiangiogenic therapy. Dissecting the molecular basis of immune modulation by anti-angiogenic therapy is therefore relevant to delineate biomarker(s) of response.

## Targeting Epigenetic Modifiers

Epigenetic modifications including DNA methylation and histone PTMS influence nearly all aspects of gliomagenesis, progression, and recurrence. Epigenetic modifications in solid tumors are gaining relevance as biomarkers and drug targets (Romani et al.). Although therapies targeting epigenetic regulators or chromatin remodeling complexes remain at early stages of development, the DNA methylation studies have helped delineate MGMT promoter hypermethylation as a robust biomarker for TMZ-based chemotherapy. Encouraged with the success of HDAC inhibitors (HDACi) in hematologic malignancies, HDACi therapies have been explored for GBM and DIPG. However, despite promising results in preclinical models, success of HDACi in clinical trials of GBM and DIPG has been modest. Radio-sensitizing effects of HDACi Panobinostat and valporic acid in phase-I clinical trials appear to be promising, but more studies are needed to support further development. BET inhibitors and EZH2 inhibitors are other epigenetic modifiers recently entered in clinical trials in GBM. While many more small molecules targeted at epigenetic pathways are on the horizon, recent discovery that majority of DIPG tumors harbor H3K27M histone protein mutation that causes global loss of H3K27me3, was of particular interest because pharmacologic restoration of H3K27me3 levels by GSK-J4, a prototype inhibitor against the H3K27me3 demethylase JMJD3, has shown excellent anti-tumor activity. However, clinical trials employing GSK-J4 have yet to be launched. Besides traditional epigenetic machinery, neomorphic IDH1 mutations result in production of 2-hydroxy glutarate, which is a strong epigenetic modulator. Targeting mutant IDH1 with IDH1-inhibitors has shown promising results in hematological malignancies and opens the way for clinical testing in GBM and low-grade gliomas harboring IDH1 mutations.

## Inhibitors of DNA Repair

Dysregulation of DNA repair pathways in tumor cells undermines the benefit of genotoxic therapies. Therefore, targeting DNA repair pathways is a rational strategy to improve the response to standard chemo-radiation therapy in GBM and DIPG. Progress has been made in understanding pathways of the DNA repair involved in resistance to chemo-radiation, leading to the discovery of range of druggable targets including MGMT and PARP. Therapeutic strategies aiming to improve response to TMZ using inhibitors of MGMT was discontinued due to severe myelosuppression in patients (Romani et al.). Since PARP plays pleiotropic role in DNA damage repair mechanisms, PARP-inhibitors (PARPi) have emerged as promising sensitizing strategy. After disappointing results from early clinical trials in recurrent GBM, and reports of limited *in vivo* sensitizing effects of PARP inhibition in TMZ-resistant GBM, several new clinical trials have been launched to evaluate PARP inhibitors in newly diagnosed GBM (Gupta et al.). Some of these trials have integrated MGMT promoter methylation as biomarker to distinguish TMZ-sensitive population. While outcome from ongoing clinical trials will determine the future of PARPi in GBM, Gupta et al. have described variables that may influence the success of PARPi in GBM.

## Re-purposing Drugs Known to Cross Blood Brain-Barrier (BBB)

FDA-approved drugs with evidence of penetration into the central nervous system (CNS) have potential as chemo-sensitizing strategy. Harder et al. report that propentofylline, previously tested in patients with vascular dementia and Alzheimer's disease suppresses pro-tumorigenic functions of microglia by targeting TROY, an orphan receptor in the Tumor Necrosis Factor Receptor (TNFR) signaling. Similarly, pimozide, an antidepressant and antipsychotic drug, and the chlorpromazine, an antipsychotic drug, inhibit multiple pro-tumorigenic activities in GBM cells (Harder et al.). Interestingly, clinical data supports chloroquine as sensitizer of standard chemo-radiation in GBM. However, in light of reports suggesting that autophagy-inhibiting effect of chloroquine is largely dispensable for tumor suppression, understanding autophagy-independent activities of chloroquine may help define potential biomarkers (Weyerhäuser et al.). The anti-diabetic biguanide class of drugs (including metformin) is interesting because biguanides selectively inhibit chloride intracellular channel1 (CLIC1), which is an emerging prognostic and predictive biomarker, as well as a promising therapeutic target in GBM (Barbieri et al.). However, repurposing of these drugs for the treatment of GBM will require optimization of cancer-relevant regimen and better mechanistic understanding.

## Targeted Immunotherapy

Immunotherapy is one of the most promising new cancer treatment approaches, and the recent reports challenging the long held opinion that CNS is an “immune privileged site” led to investigations aimed at boosting host immunity. While the immunosuppressive tumor microenvironment prevents immune response in GBM, manipulating the host immune system using immune check point blockade (ICBs) is considered a reasoned strategy. As summarized by Romani et al., clinical trials evaluating ICBs as single agent or in various combinations with standard cytotoxic, targeted or other immunological therapies are ongoing. Although results of a large phase III trial are disappointing, but not surprising given the fact that gliomas carry a substantially low tumor mutational burden, an important feature associated with anti-tumor immunogenicity. Results of some phase I/II trials of ICBs combined with Bevacizumab and radiotherapy (RT) appear encouraging, which is likely due to enhanced immune response with RT and/or bevacizumab (Malo et al.). However, further studies may be required to analyze effects of RT, which can be an independent synergistic facilitator of response to immunotherapy Rajani et al., especially in genetically unstable tumors, where enhanced TMB with RT is possible. In context of recurrent tumors, where RT is precluded, using oncolytic agents in combination with ICBs may facilitate antitumor response. The impacts of prior brain RT in recurrent tumors is poorly understood, though increasing evidence suggest that RT-induced changes in brain may contribute to recurrence and aggressiveness of GBM (Gupta and Burns). Whether containment of CNS injury responses in brain after RT improves response to ICB therapy has to be carefully assessed. Epigenetic mechanisms by regulating expression of PD-1 and PD-L1, can modulate response to ICBs (Chin et al.). Therefore, targeting epigenetic pathways involved in PD-1 and PD-L1 upregulation can promote anti-tumor immunity and may synergize immunotherapy drugs (Chin et al.).

## Adoptive Immunotherapy

Defective antigen processing, T-cell receptor signaling, co-stimulatory signaling or immune-surveillance capacity of natural killer (NK) cells may disrupt immune response even in presence of adequate TMB. Adoptive transfer of immune cells, trained or modified to attack cancer cells, has emerged as an attractive immunotherapy strategy. In this line of therapeutics, dendritic cell (DC) vaccines, activated NK-cells and chimeric antigen receptor (CAR) expressing T cells (CAR-T) or CAR expressing NK cells (CAR-NK) are under intense investigation.

### DC Vaccines

DCs being the most prominent antigen presenting cells (APCs) are essential for sustained T cell and NK cell response. DC vaccines involve autologous transfer of DCs incubated with glioma stem cells or mixture of GBM associated peptides or tumor-specific peptide such as EGFRvIII extracellular domain. Early stage clinical trials of DC vaccines have yielded promising results in select groups of patients with GBM but have not met primary endpoint to extend overall survival time. Whether combining DC vaccines with the ICBs, improves overall response remains to be tested (Jain; Romani et al.; Rajani et al.).

### CAR-T Cells

Since the use of genetically engineered T-cells expressing CARs (fusing extracellular antigen recognition domain directed against tumor specific antigens with transmembrane and intracellular domain of T-cell receptor), has been FDA approved for hematologic malignancies. A number of preclinical and clinical studies have been evaluating this strategy in solid tumors. At least 3 independent phase-I trials have demonstrated feasibility, safety, and encouraging signs of efficacy of CART cells directed against EGFRVIII, HER2, or IL13Ra2, well-known surface antigens in subgroups of GBM. While promising results have generated enthusiasm for CAR-T cell therapy of brain tumors, expanded search for CAR targets, improved trafficking and optimization of dose, frequency and schedule of administration, will be key to advancement of CAR-T cell therapy. Considering low engraftment, lack of proliferation or effector function of T-cells in brain tumor microenvironment, CAR-T cell therapy alone may not be sufficient. Combining CAR-T cell therapy with ICBs, oncolytic agents and/or lymphodepleting chemotherapy should be a more comprehensive and efficacious approach.

### NK and CAR-NK Cells

NK cells in immunosuppressive environment of brain tumors lack immune-surveillance capacity. Therefore, transferring *ex-vivo* activated NK cells appears to be a promising approach to brain tumors. In a phase I clinical trials, autologous transplantation of *ex-vivo* activated NK cells (with IL-2 or IL-15) into the resection cavity of GBM patients, has shown anti-tumor activity. Similarly, allogeneic transplantation with continuously expanding NK-92, a constitutively active human NK cell line, has been safely applied that showed clinical response in a subset of patients. Similar to CAR-T cells, NK cells engineered to express CARs have been developed for targeted lysis of cancer cells. As a proof of principle study robust antitumor efficacy of NK-92 cells expressing an ErbB2-specific CAR have previously been demonstrated in syngeneic mouse models. While activated NK or CAR-NK cells appear to obviate several challenges of DC vaccine and/or CART cell therapy, the ongoing clinical trials will ultimately determine the fate of NK or CAR-NK cell-based treatments for human gliomas.

## Improvements in Drug Delivery

Exclusion of toxins from entering the brain is one unique tissue BBB (Harder et al.; Himes et al.). Despite tumor vasculature being underdeveloped and leaky, throughout history, one of the leading challenges in treating brain tumors has been delivery of drugs past the BBB. For DIPG tumors, this might be harder, as there is evidence indicating that the BBB is even more privileged. Finding BBB-penetrating drugs, which can maintain effective steady state concentrations without causing toxicity to normal tissue, is vital but serious limitation to the development of targeted therapies. Developing new and safer methods of drug delivery to disrupt or bypass the BBB is an area of intensive research and multiple methods including convection enhanced delivery (CED), focused ultrasound (FUS), vasoactive peptides, osmotic agents, and polymeric nanoparticles encapsulation are being developed (Harder et al.).

Himes et al. demonstrate that despite technical challenges, placement of CED catheters into the brainstem of small animals is safe. This is in line with the phase I safety trial in patients with DIPG tumors, where CED of the radionuclide [^124^I]-8H9 was well-tolerated. Several ongoing clinical trials continue to investigate CED of various promising drug formulations for DIPG and GBM treatment that brings hope to patients. However, developing CED as a routine procedure is an ongoing challenge that requires further refinements in hardware technology and the understanding of CED pharmacology. Although preclinical and clinical studies of CED continue to enhance the pipeline of targeted agents for both DIPG and GBM, the invasive and highly technical nature of the procedure remains an obstacle.

Macromolecular drug delivery systems, such as liposomes and polymers, increase efficacy, stability, and plasma half-life of anticancer drugs while reducing toxicity to healthy tissues. Drug delivery through macromolecular carriers mostly relies on the passive targeting via the enhanced permeability and retention effect. Raucher et al. describe the use of macromolecular carriers that deliver and/or release drugs in response to internal or external stimuli. Additional studies are required to understand the pharmacology of macromolecular carriers, and refine assays to precisely measure toxicity of these promising macromolecular carriers.

Tumor-tropic properties of neural stem cells (NSCs) permit their use as delivery vehicles to selectively target therapeutic gene products to brain tumor cells (Gutova et al.). The clinical trials to date with the allogeneic, clonal HB1.F3.CD21 NSC line have demonstrated safety, injections through intracranial tracts (ICT) are technically challenging. Gutova et al. have developed intracerebral/ventricular (IVEN) method of delivery to overcome the challenges in ICT route of delivery. NSCs delivered by IVEN route in mice with intracranial GBM xenografts, migrated to contralateral brain and localized within tumors. Robust migration of clinically relevant HB1.F3.CD21 NSCs toward invasive tumors shows the feasibility of IVEN to deliver NSCs in to brain tumors and is likely to have impact on gene therapy based treatments of brain tumors.

## Conclusions and Future Perspectives

The lack of bioactive brain penetrant-targeted molecules and inadequate considerations to genomic/molecular features of tumors may be partly responsible to systemic failure of targeted therapies in clinical trials. Although all targeted agents may have gone through preclinical testing to justify evaluation in clinical trials, repeated clinical failures of novel investigational drugs highlight the importance of comprehensive preclinical assessment of brain pharmacokinetics and efficacy evaluation involving genetically engineered animal models or larger panels of orthotopically implanted PDXs rather than justifying clinical trials based on *in vitro* cytotoxicity data or *in vivo* efficacy evaluation in limited number of xenografts established from cell lines. Integration of technological advances in drug delivery, patient stratification based on matching molecular characteristics and robust prognostic and predictive biomarkers in modern clinical trial designs will be crucial to successful translation of promising targeted therapies.

## Author Contributions

SG: conception, design, and writing. SK, DD, and JS: reviewed and helped to revise the manuscript.

### Conflict of Interest

The authors declare that the research was conducted in the absence of any commercial or financial relationships that could be construed as a potential conflict of interest.

